# European Regulatory Framework and Safety Assessment of Food-Related Bioactive Compounds

**DOI:** 10.3390/nu12030613

**Published:** 2020-02-26

**Authors:** Ariane Vettorazzi, Adela López de Cerain, Julen Sanz-Serrano, Ana G. Gil, Amaya Azqueta

**Affiliations:** 1 Department of Pharmacology and Toxicology, University of Navarra, 31008 Pamplona, Spain; acerain@unav.es (A.L.d.C.); jsanz.3@alumni.unav.es (J.S.-S.); agil@unav.es (A.G.G.); amazqueta@unav.es (A.A.); 2 IdiSNA, Navarra Institute for Health Research, 31008 Pamplona, Spain

**Keywords:** bioactive compound, food supplement, novel food, botanicals, European regulation, European Food Safety Authority (EFSA), safety assessment, toxicity, nutraceuticals, functional food, functional ingredient

## Abstract

A great variety of functional foods, nutraceuticals, or foods with bioactive compounds are provided nowadays to consumers. Aware of the importance of the safety aspects, the food industry has to comply with different legal requirements around the world. In this review, the European regulatory framework for food-related bioactive compounds is summarized. The term ‘bioactive compound’ is not defined in the European regulations, however, since they can be part of food supplements, fortified foods, or novel food, they are included within the legal requirements of those corresponding types of foods or supplements. Lists of authorized compounds/foods appear in the correspondent regulations, however, when a new compound/food is going to be launched into the market, its safety assessment is essential. Although the responsibility for the safety of these compounds/foods lies with the food business operator placing the product on the market, the European Food Safety Authority (EFSA) carries out scientific evaluations to assess the risks for human health. To facilitate this procedure, different guidelines exist at the European level to explain the tier toxicity testing approach to be considered. This approach divides the evaluation into four areas: (a) toxicokinetics; (b) genotoxicity; (c) subchronic and chronic toxicity and carcinogenicity; and (d) reproductive and developmental toxicity.

## 1. Introduction

Over the last few decades, there has been a rapid increase in the knowledge of the biochemical and physiological cellular functions of human nutrients; in addition, the recommendations regarding macronutrients, micronutrients, and essential nutrients intake for a healthy status have increased. Moreover, the social perception of nutrition habits and physical activity, and their impact on the present and future health has rapidly evolved. Thus, the scientific knowledge and the consciousness of the importance of nutrition to prevent disease have increased the offer of foods with known healthy properties. A great variety of functional foods or foods with bioactive compounds or nutraceuticals can nowadays be easily found in the global market. In this global scenario, the food industry, which is aware of the importance of the safety aspects, has to comply with the legal requirements of regulatory frameworks that can be very different between countries.

The objective of this article was to review the safety regulatory aspects in the European Union, and summarize the toxicity evaluation and safety assessment of bioactive compounds that can be found in food. First, clarification of the different terms used for food compounds that have a beneficial healthy effect is provided.

“Bioactive compounds”, “nutraceuticals”, and “functional foods” are some of the terms widely used in the food context by both the industries and consumers, however, the boundaries between them all are not clear and are very often used interchangeably or with different nuances. Some of the definitions found in the recent literature are shown in Table 1. As can be observed, although these terms are commonly used worldwide, they are not internationally agreed upon and there is no consensus on their meaning. For further details about the inconsistencies in the definitions of these terms, the readers are referred to the article by Aronson [1]. 

The most basic definition of a bioactive compound found in many language dictionaries is “having or producing an effect on a living organism”. However, this broad definition is not precise enough for the current safety assessment framework and regulatory standards for human health. Indeed, it does not specify: (i) if the effect is beneficial or deleterious, (ii) in case of being beneficial, if the type of effect is pharmacological or nutritional, and (iii) the final form or vehicle in which the bioactive is present (in food or in a pre-formed presentation). The first point mentioned (i) points directly to safety evaluation issues, which is the cornerstone of EU legislation on food, while the type of effect (ii) (pharmacological versus nutritional) is a key point to differentiate between “pharmaceuticals” and “any bioactive compound present in food”, independently of the final form in which they are vehiculized (iii). 

Indeed, many bioactive compounds obtained from plants, fungi, or vegetables have been used as drugs for millennia, and many of them have become medicinal drugs, and so evaluated and commercialized as such. Therefore, at the European level, they are under the scope of Directive 2001/83/EC (and its amendments) [12] and are defined as medicinal drugs: “Any substance or combination of substances presented as having properties for treating or preventing disease in human beings; or any substance or combination of substance which may be used in or administered to human beings either with a view to restoring, correcting or modifying physiological functions by exerting a pharmacological, immunological or metabolic action, or to making a medical diagnosis”. 

Another important aspect in the definitions proposed for bioactive compounds (Table 1) is that they occur in “food”. Under the EU general food law (Regulation (EC) No. 178/2002) [13], food is defined as “any substance or product, whether processed, partially processed or unprocessed, intended to be, or reasonably expected to be ingested by humans”. This regulation clearly states that medicinal products are not included in this definition. Thus, any bioactive compound, present or intentionally added to food, might fall within this regulation. Unfortunately, neither of the terms “bioactive compound”, “nutraceutical”, nor “functional food” are defined, as such, within the current EU food regulatory framework. However, a wide range of nutrients and other ingredients (including “bioactive compounds”) might be present or added to different groups of foods including, but not limited to, vitamins, minerals, amino acids, essential fatty acids, fiber, and various plants and herbal extracts. 

Therefore, the specific aims of the present review are: (i) to compile the terms associated to food-related bioactive compounds (independently of whether they are naturally occurring or intentionally added) under the European regulatory framework (Section 1), and (ii) to explain the assessment needed to be done in order to comply with the European safety standards (Section 2). The present review does not focus on substances (bioactive or not) with technological functions such as additives or pesticides or with pharmacological effects such as medicinal drugs.

## 2. European Regulatory Framework 

At the European level, the general principles and requirements of food law, together with the establishment of the European Food Safety Authority (EFSA) and the basis for food safety, are laid down in the EU General Food Law (Regulation (EC) No. 178/2002) [13]. This regulation also embodies the “precautionary principle”, the heart of decision-making related with food-related matters when uncertainties are present. The readers are referred to the report by van der Roest et al. (2003) [14] for a review of the interpretation of this principle.

As previously mentioned, nutraceuticals, food/functional, or bioactive compounds are not regulated at the European level as such. However, any kind of bioactive compound susceptible for inclusion in food may fall under one (or more) regulation(s) as shown in Table 2 and further explained below. 

At the EU level, one of the terms that appears specifically defined is food supplement and is defined under Directive 2002/46/EC [15] as: “foodstuffs the purpose of which is to supplement the normal diet and which are concentrated sources of nutrients or other substances with a nutritional or physiological effect, alone or in combination, marketed in dose form, namely forms such as capsules, pastilles, tablets, pills and other similar forms, sachets of powder, ampoules of liquids, drop dispensing bottles, and other similar forms of liquids and powders designed to be taken in measured small unit quantities”. Their main characteristics are that they are marketed under “dose” form (e.g., capsule) with the aim of correcting nutritional deficiencies, maintaining the intake of nutrients, or supporting certain physiological functions. Thus, according to this definition, food supplements are not medicinal products and as such cannot exert a pharmacological, immunological, or metabolic action. Therefore, their use is not intended to treat or prevent diseases in humans or to modify physiological functions [20].

Directive 2002/46/EC [15] represents a first step in the harmonization process of bioactive compounds, however, it focuses only on vitamins and minerals, providing both harmonized lists of the vitamins and mineral substances (and their sources) used in the manufacture of food supplements and the labelling requirements for these products. Thus, other substances susceptible of being present in food supplements are not fully covered by this Directive. The use of compounds other than minerals and vitamins might be regulated by national rules (mainly within specific Royal Decrees for (i) nutrients (vitamins and minerals), (ii) plants, and (iii) other substances) or may be authorized under other EU legislations such as the regulations (explained below) related to substances for the fortification of food, foods for specific groups, and novel foods. 

For instance, in relation to the fortification of foods, Regulation (EC) No. 1925/2006 [16] harmonizes the provisions regarding the addition of vitamins and minerals and of certain other substances to foods. In this case, the nutrients or ingredients are added to food in order to “enrich” or “fortify” the food in question to add or emphasize particular nutritional characteristics [21]. Thus, the final presentation is that of the enriched food and does not have a pre-packaged form as in the case of food supplements. Regulation (EC) No. 1925/2006 [16] includes substances other than vitamins and minerals (including trace elements), for example, essential fatty acids, fiber, and various plants and herbal extracts. It is worth mentioning that this regulation concerns all foodstuffs, and thus, it also affects food supplements. However, it specifically states that the provisions regarding vitamins and mineral (Annexes 1 and 2) shall not apply to food supplements covered by Directive 2002/46/EC [15]. Annex 3 contains a list of substances other than the vitamins and minerals whose use in foods is prohibited, restricted, or under scrutiny. Some prohibited substances listed in Annex 3 are, for example, Ephedra herb and Yohimbe bark or their preparations from Ephedra species or Pausinystalia yohimbe, respectively. These restrictions, thus, apply to all foodstuffs (including food supplements). 

Another regulation that should be also taken into account is Regulation (EU) 609/2013 [17] on Foods for Specific Groups (FSG). This Regulation establishes compositional and information requirements for different food categories: (i) infant formula and follow-on formula, (ii) processed cereal-based food and baby food, (iii) food for special medical purposes, and (iv) total diet replacement for weight control. Moreover, it also establishes a single list of substances (and the rules applicable to the updating of that list) that may be added to one or more of the categories of food previously mentioned and lays down the rules applicable to the updating of that list. Thus, any substance (and thus any bioactive compound) that might be added to this type of food should also comply with this regulation. This list includes minerals and vitamins but also amino acids, carnitine and taurine, nucleotides, and choline and inositol. It should be mentioned that delegated acts such as Regulation (EU) 2016/127 [22], Regulation (EU) 2016/128 [23], or (EU) 2017/1798 [24] that provide specific composition and labeling rules for the different food categories are progressively replacing the previous legal framework.

Finally, Regulation (EU) No. 2015/2283 [18] on Novel Foods affects any kind of food that has not been consumed to a significant degree by humans in the EU before 15 May 1997 (date of when the first Regulation on novel food came into force). Thus, this regulation covers a wide range of cases such as new foods, food from new sources, new substances used in food as well as new ways and technologies for producing food [25]. Some bioactive compounds obtained from or added to food may also fall within this “novel food” category. Some of the examples given by EU as novel foods are new sources of vitamin K (menaquinone) or extracts from existing food (Antarctic Krill oil rich in phospholipids from Euphausia superba), agricultural products from third countries (chia seeds, noni fruit juice), or food derived from new production processes (UV-treated food (milk, bread, mushrooms, and yeast). More precisely, some of the specific cases that may affect bioactive compounds under the Novel Foods regulation are: (i) vitamins, minerals and other substances used in accordance with Directive 2002/46/EC [15], Regulation (EC) No. 1925/2006 [16], or Regulation (EU) No. 609/2013 [17], where: (a) a production process not used for food production within the Union before 15 May 1997 has been applied; or (b) they contain or consist of engineered nanomaterials; (ii) food used exclusively in food supplements within the Union before 15 May 1997, where it is intended to be used in foods other than food supplements as defined in Directive 2002/46/EC [15]. It should be mentioned that the list of novel foods authorized for the EU market, their conditions of use (including maximum levels), labeling, and other requirements as well as detailed specifications are provided in the Commission implementing Regulation EU 2017/2470 [19]. 

It is worth mentioning that many of the bioactive compounds used in food (especially as food supplements) are naturally occurring substances called “botanicals or derived preparations”. These “botanical” products are becoming popular and widely available on the EU market (in pharmacies, supermarkets, specialized shops, or online) and are labelled as “natural”, together with many claims regarding their possible health effects. Many of these bioactive compounds have a long history of use, but this is not always the case, and some concerns might exist regarding their safety and quality [26]. As food supplements, they have to comply with the general food law (Regulation (EC) No. 178/2002) [13], however, there is still no centralized legal authorization procedure at the EU level for the use of these “botanicals” in food. In order, to give some harmonized information and help in the risk assessment process, the EFSA has created a database of botanicals that might be used in food [27], but is not exhaustive and does not have any legal force. 

Finally, other important legal frameworks, but out of the specific scope of the present review, also affect some of the foods previously mentioned. This is the case, for example, of any food containing additives or bioactive compounds with technological functions that shall comply with Regulation (EC) 1333/2008 [28] on food additives, or of any food claiming any nutritional or health effects. In this case, the claim should also comply with Regulation (EC) No. 1924/2006 [29] on nutrition and health claims. 

Overall, at the EU level, bioactive compounds aimed to be used in food could fall under one (or several) of the regulations affecting: (i) food supplements, (ii) substance for the fortification of food, (iii) food for specific groups, and (iv) novel foods. On the other hand, depending on the type of bioactive compound, they can be classified as (i) vitamins and minerals, (ii) other substances, or (iii) botanicals. 

It is important to note that the same bioactive ingredient (for example, vitamin C) might fall in one or other regulation(s) depending on the final intention of use. In general, falling within the scope of one or other regulation(s) will depend on the final presentation (capsule/pill versus foodstuff), the functionality of the bioactive compound (additives—out of the scope of the present review—aimed at having a food technological function versus food supplements or fortified food aimed at correcting nutritional deficiencies), and history of consumption of the food at the EU level (food consumed significantly prior to May 1997 versus new foods). However, it should also be noted that depending on the type of bioactive compound, the regulation is more or less developed. For example, within the food supplements framework, vitamins and minerals are more clearly regulated than the “other substances” or “plant extracts/botanicals”.

## 3. Safety Assessment and Toxicological Evaluation

As mentioned in the previous section, there are lists of bioactive compounds that have already been approved for use in foods. The Food Supplements Regulation provides lists of vitamins and minerals and vitamin and mineral substances that may be used in the production of food supplements (Directive 2002/46/EC) [15]. The Regulation for the Fortification of Food provides lists of vitamins and minerals and vitamin formulations and mineral substances that may be added to food as well as a list of other types of compounds that are prohibited, restricted, or under scrutiny (Regulation (EC) No. 1925/2006) [16]. In the same way, the Novel Food Regulation also contains a list of the novel foods authorized (regulation (EC) No. 2015/2283) [18]. These lists have been established by the scientific risk assessment performed and published by the former Scientific Committee on Food (SCF) of the European Commission (EC) or the European Food Safety Authority (EFSA) and based on generally accepted scientific data. 

The responsibility for the safety of food supplements, fortified foods, or novel foods lies with the food business operator placing the product on the market. In general, regulations mentioned in the previous section do not contain strategies or guidelines related to the toxicological evaluation; they only specify that the safety of each type of food launched into the market should be guaranteed. In this regard, the SCF and, later on, the EFSA, have published scientific opinions in the form of guidance to carry out the safety assessment of such substances. These guidelines assist the applicant in organizing the information in order to prepare a well-structured application to demonstrate the safety of the new compound/food. This will facilitate the EFSA to carry out its evaluation. 

In this section, the safety assessment process is explained, mainly focusing on the toxicological assessment according to the final use or characteristics of the bioactive compound: source of vitamins or minerals for food supplements and fortified food (Section 3.1), novel foods (Section 3.2), or botanicals (Section 3.3). The specific safety assessment of food for specific groups focused on the nutritional criteria will not be covered in the present section. Finally, Section 3.4 focuses on the specific details of the toxicological evaluation that is normally required to be carried out in all cases. 

### 3.1. Source of Vitamins and Minerals for Food Supplements and Fortified Foods

As mentioned in the introduction, there are lists of vitamin and minerals and sources of vitamins and minerals permitted for used in food supplements and fortified food (Directive 2002/46/EC, Regulation (EC) No. 1925/2006) [15,16]. 

Regarding the nutrient itself, the SCF and EFSA performed an evaluation of the vitamins and minerals with the aim of establishing tolerable upper intake levels (ULs) for different groups of population; ULs being the highest dose in terms of chronic daily intake that is likely not to induce an adverse effect in humans. Like other chemical substances, vitamins and minerals may have adverse effects if consumed in excessive amounts, and thus the same principles of toxicological characterization apply to these micronutrients like other food chemicals. The specific principles for evaluation of the adverse effects of micronutrients in humans and for establishing upper levels of intake are explained in the SCF scientific opinion ”Guidelines of the scientific committee on food for the development of tolerable upper intake levels for vitamins and minerals” [30]. One important point to highlight when evaluating the toxicity of vitamins and minerals is that, as essential components of the human diet, it is necessary to take into account that there is a (lower) level of intake below which the risk of deficiency conditions or sub-optimal functioning arises. The established ULs support the EC in establishing the maximum limits. However, due to the complexity of this issue, the minimum and maximum levels of vitamins and minerals used in food supplements and fortified foods have been consulted extensively by the EC with the Members States and interested stakeholders. At the moment, no regulatory proposal has been presented yet. 

In relation to the source of these nutrients, when a company wants to launch a new source of vitamins or minerals on the market that is not included in the permitted lists, they have to submit an application to the European Commission, who may request that the EFSA perform the corresponding evaluation of the safety assessment. These safety assessments are published by the EFSA as scientific opinions. The EC reviews and updates the lists, taking into account the evaluations of the EFSA. The application includes a technical dossier that should be prepared following the corresponding EFSA guideline entitled “Guidance on safety evaluation of sources of nutrients and bioavailability of nutrient from the sources” [31]. According to these guidelines, the technical dossier should contain the following scientific data: characterization of the proposed source, existing authorizations or evaluations, proposed use/s and exposure assessment (dietary exposure), toxicological data, and availability of the nutrient (in comparison with forms of the same nutrient that are already permitted in the positive list). 

The dissociation and the extent of dissociation of the source in the lumen of the human gastrointestinal tract is a key point in the evaluation of the safety assessment of a source of vitamins or minerals. Toxicological data should focus on the source and the relevant breakdown products. Existing toxicological evaluations of the resulting compounds should be included. If not available or not adequate, new data should be produced. In any case, the guidelines state that the tiered toxicity testing approach published as part of a scientific opinion entitled “Guidance for submission for food additive evaluations” should be considered [32] (for the description of the toxicity testing approach see Section 3.4.). Some specifications regarding how to follow the toxicity testing strategy depends on the dissociation of the source. 

The guidelines regarding the safety assessment of the source of vitamins and minerals include a table in which different categories of substances that can be proposed as a source of vitamins or mineral are presented. This table also contains the additional data requirements for the safety assessment, depending on the category. Within the categories, the sources of botanical origin and sources related to novel foods are included. In this case, the information given in the following sections should also be considered. 

### 3.2. Novel Foods

As mentioned previously, there is a list of novel foods authorized (Regulation (EC) No 2015/2283 and Implementing Regulation (EU) 2017/2470)) [18,19]. This list can increase with the inclusion of new authorized novel foods. The process is similar to the one described in Section 3.1. In the case of traditional foods from third countries, the safety assessment is based on a history of safe food use and the process to place the product on the market is different. The notification is sent to the EC, who will forward it to all of the Member States and EFSA. Then, if they submit reasoned-safety objections, the applicant may apply for it following the normal channel.

In 2016, the EFSA published the scientific opinion entitled “Guidance on the preparation and presentation of an application for authorization of a novel food in the context of Regulation (EU) 2015/2283” [33]. In this case, the dossier should contain the description of the novel food, the production process, the compositional data, specification, proposed uses and use levels, anticipated intake of the novel food (other potential sources of intake of the novel food), history of use of the novel food and/or its source, absorption, distribution, metabolism and excretion data (ADME), nutritional information, toxicological information, and allergenicity. In the case of traditional foods from a third country, EFSA has prepared a separate guideline for preparing and presenting a dossier [34].

The guidelines state that the tiered toxicity testing approach for food additives should also be applied [32] (for the description of the toxicity testing approach see Section 3.4.). It is important to take into account that a novel food can be a single substance, simple mixtures, and complex mixtures, but also a whole food and the toxicity evaluation is different as will be addressed later. The guidelines on novel foods [33] present the following elements to decide which toxicity studies need to be conducted: the identity, chemical structure, composition, and physico-chemical properties of the novel food; available information on previous human consumption of the novel food and its source; anticipated use(s), maximum use levels, and the resulting intakes; available kinetic data; available toxicological data on the novel food or its constituents; available human studies; available relevant information on non-food uses (e.g., cosmetics, chemicals, pharmaceuticals); and in the case of insufficient experimental data also the (quantitative) structure–activity relationship ((Q)SAR) data. Toxicological data on structurally related substances (‘read-across’) should be considered.

Botanicals can be novel foods as whole plants, compounds isolated from plants, or compounds produced from plants. In any case, the specific considerations provided by the EFSA for botanicals and botanical preparations should be taken into account (see Section 3.3). 

### 3.3. Botanicals

It is important to mention that the EU does not have a centralized authorization procedure for the use of botanicals and botanical preparations in food. Business operators are responsible for the safety of these products and must comply with the general requirements set out in the general food law (Regulation (EC) No. 178/2002).

In 2009, the EFSA published the Guideline entitled ‘Safety assessment of botanicals and botanical preparations intended for use as ingredients in food supplements’ [35] to help any organization carry out a harmonized and safe assessment of these types of compounds. This guidance is focused on botanicals and botanical preparations that are intended for use in food supplements, although it also states that, in principle, it is applicable to other uses in the food and feed areas. 

The following data are required to perform the safety assessment of botanicals and botanical preparations: technical data as the identity and nature of the source material, manufacturing process, chemical composition, specifications (concentrations of major groups of constituents present in the botanical preparation and maximum levels for possible contaminants), stability, proposed uses and use levels and information on existing assessments; data about exposure (extent and duration); and toxicological assessment.

For the toxicological assessment of botanicals and botanical preparations, the EFSA has proposed an approach consisting of two levels: (1) safety assessment based on available knowledge (level A), and (2) safety assessment including newly generated data (level B). Regarding level A, available data from large population groups exposed to the ingredient (at a known level) can allow for conclusions regarding the absence of adverse effects. Actually, the EFSA has published the ‘Scientific Opinion on a Qualified Presumption of Safety (QPS) approach for the safety assessment of botanicals and botanical preparations’ [36] to be used as an extension of the guidelines on botanicals and botanical preparations. Regarding level B, the toxicity testing approach suggested is the one included in the scientific opinion prepared by the EFSA in 2012 for the submission of food additive evaluations [32]. 

Due to the great amount of botanicals and their bioactivity (and toxicity), the EC has not produced a list of authorized ones. However, the EFSA has created a “Compendium of Botanicals” that have been reported to contain substances that may be of health concern when used in food or food supplements [27]. These are botanicals that have been reported to contain toxic, addictive, psychotropic, or other substances that may be of concern. This compendium has no legal or regulatory force. In fact, some of the botanicals in the compendium are ones prohibited under the regulation about the fortification of food (Regulation (EC) No. 1925/2006) [16] (i.e., Ephedra herb and its preparations originating from the Ephedra species and Yohimbe bark and its preparations originating from Yohimbe [Pausinystalia yohimbe (K. Schum) Pierre ex Beille]. The compendium of botanicals as well as the EFSA Chemical Hazard Database ([37], see the link to the database) are recommended as tools to find possible substances of concern for novel foods [33].

For botanical substances or botanical preparations going to be used as a source of nutrients or as a novel food, the corresponding process to put them on the market and perform the safety assessment should be followed (see Section 3.1 and Section 3.2). 

### 3.4. Toxicity Evaluation

The toxicity evaluation is a key point in all safety assessments. The EFSA guidelines produced to perform the safety assessment of the source of vitamins and minerals, novel foods, or botanicals suggest following the tier toxicity testing approach proposed for food additives in 2012 [32] (Table 3). This approach divides the evaluation into four core areas: toxicokinetics; genotoxicity; toxicity encompassing subchronic, chronic toxicity, and carcinogenicity; and reproductive and developmental toxicity.

Toxicokinetic evaluation provides data on the systemic exposure to the chemical and its metabolites as well as on the process involved in their absorption, distribution, metabolism, and excretion (ADME). It gives valuable information for the selection of the appropriate species and doses for toxicity testing and for the interpretation of the toxicity studies. Comparison of the animal internal dose to human exposure is very relevant for the safety assessment and both in vitro and in vivo techniques can be used.

Genotoxicity studies the effect of chemicals on the DNA. It is known that genetic alterations are associated with serious health effects such as cancer and degenerative conditions. Moreover, genetic alteration in germ cells may induce abortions, infertility, or heritable damage. In genotoxicity testing, both the formation of point and chromosomal mutations, both structural and numerical, are evaluated using different in vitro and in vivo techniques. 

Toxicity studies are performed to assess changes in blood, urine, and clinical biochemistry parameters as well as gross and histopathological changes in organs and tissues following a prolonged exposure of animals to the test material. These studies may also provide information on neurofunctional and neurobehavioral effects. Subchronic toxicity studies (i.e., at least 90 days of daily exposure) establish the main toxicological profile of the substance and provides information on the target organs and tissues affected, on the nature and severity of any effects, and on the dose-response relationships. Moreover, it allows the determination of very important toxicological parameters such as the non-observed adverse effect level (NOAEL), which is the highest dose at which adverse effects are not detected. Subchronic toxicity studies allow for the estimation of the dose to be used in chronic studies including carcinogenicity studies; the highest dose levels in these studies should induce some toxic effect. In carcinogenicity studies, animals are exposed to the test compound daily for two years and the endpoint is the formation of tumors.

Reproductive toxicity and developmental toxicity studies are performed in animals in order to obtain information on the effects on the reproduction (e.g., fertility, pregnancy, prenatal and postnatal survival, growth, functional and behavioral development of the offspring and reproductive capacity of the offspring) and on prenatal development (e.g., lethality, toxic effects on the embryo and fetus, teratogenicity, and sex ratio). Exposure can occur prenatally via the mother during the pregnancy and postnatally via maternal milk. 

Moreover the toxicity approach consists of three tiers: tier 1 testing includes the minimal data required for all compounds; tier 2 applies to the compounds that are absorbed or demonstrate toxicity or genotoxicity in tier 1; and tier 3 is performed on a case by case basis to study specific endpoints, depending on the results obtained in tier 2. Higher tier testing may be required in some cases. Table 3 shows the toxicity testing approach proposed for food additives. 

As can be observed in Table 3, studies should be conducted using the test methods described in the Organization for Economic Co-operation and Development (OECD) [52]. These are internationally agreed test guidelines to perform test methods with the aim of assessing the potential effects of chemicals on human health and the environment. These are divided into five sections: physical chemical properties; effects on biotic systems; environmental fate and behavior; health effects; and others. The guidelines compiled in the section about the health effects are the ones used for the toxicity evaluation of the source of vitamins and minerals, novel foods, or botanicals. Moreover, all studies should be performed on the basis of Good Laboratory Practice (GLP). 

In in vivo studies, substances should normally be administered via the oral route and the highest dose should normally not exceed 5% of the diet in order to avoid nutritional imbalances. Deviation from the strategy or test proposed or protocols should be justified. All relevant knowledge on the novel foods should be considered in order to make decisions on whether and which toxicity studies are necessary. Human data from epidemiological studies, if available, should also be included for the evaluation. The ‘presumption of safety’ can be applied in some circumstances.

Testing engineered nanomaterials (ENMs), which are also used as bioactive compounds or as carriers, may require the modification of some toxicity testing methods. According to the EFSA recommendation of the safety assessment of additives [32], the EFSA scientific opinion on testing this type of material should be followed [53]. Readers should note that in 2018, the EFSA published a revised version taking into account the new developments in the field [54].

The scientific opinion regarding the preparation and presentation of an application for authorization of a novel food [33] also specifies that additional toxicity studies may be required in order to examine specific biological processes. These can include immunotoxicity, hypersensitivity, and food intolerance, among others. 

As it happens in the case of food additives, the study of the ADME of complex mixtures or whole foods should be performed with the toxicologically relevant constituents (i.e., major components and other components with known or demonstrable biological or toxicological activity) [32,33]. However, special considerations regarding the dose selection are required when testing the toxicity of whole foods; the guidance prepared by the EFSA to conduct subchronic oral toxicity with this type of material should be consulted [55]. Genotoxicity studies of complex mixtures or whole foods should be focused on specific constituents [33]. On the other hand, the matrix effect should be considered; kinetics and toxicity can be modified by the matrix in which a certain compound is present. A matrix effect should be evaluated on a case by case basis [32,35].

It is worth mentioning that when a potential toxicological effect is identified, this should be discussed, taking into account the anticipated/calculated intake of the substance/food.

Overall, the safety assessment of a bioactive compound (including botanicals) should be done by taking into account if it will be part of a food supplement or fortified food, or novel food. In all cases, the toxicity testing approach follows the general principles of toxicity characterization described for testing additives in food (Section 3.4). 

## Figures and Tables

**Table 1:** Non- exhaustive list of definitions found in the published literature.

Term	Definition	Reference
Bioactive compound	**1**. Bioactive food components are constituents in foods or dietary supplements, other than those needed to meet basic human nutritional needs, which are responsible for changes in health status	[2]
	**2**. A type of chemical found in small amounts in plants and certain foods (such as fruits, vegetables, nuts, oils, and whole grains). Bioactive compounds have actions in the body that may promote good health. They are being studied in the prevention of cancer, heart disease, and other diseases.	[3]
	**3**. Bioactives are compounds that typically occur in small quantities in food and can be beneficial for health. Unlike essential macro- and micronutrients (such as fats, carbohydrates, protein, vitamins and minerals) they are not essential for life and the body can function properly without them.	[4]
Nutraceutical	**1**. Any substance that is a food or a part of a food and provides medical or health benefits including the prevention and treatment of disease. Such products may range from isolated nutrients, dietary supplements, and specific diets to genetically engineered designer foods, herbal products, and processed foods such as cereals, soups, and beverages. It is important to note that this definition applies to all categories of food and parts of food, ranging from dietary supplements such as folic acid used for the prevention of spina bifida, to chicken soup, taken to lessen the discomfort of the common cold. This definition also includes a bio-engineered designer vegetable food, rich in antioxidant ingredients, and a stimulant functional food or pharmafood.	[5]
	**2**. A nutraceutical is a product isolated or purified from foods that is generally sold in medicinal forms not usually associated with food. A nutraceutical is demonstrated to have a physiological benefit or provide protection against chronic disease.	[6]
	**3**. Nutraceuticals differ from dietary supplements in the following aspects: Nutraceuticals must not only supplement the diet but should also aid in the prevention and/or treatment of disease and/or disorder. Nutraceuticals are represented for use as a conventional food or as the sole item of meal or diet.	[7] (definition of dietary supplements according to US Dietary Supplement Health and Education Act of 1994)
	**4**. Nutraceutical, a dietary supplement, has a potential to deliver a concentrated form of a presumed bioactive agent from a food, presented in a non-food matrix and used with the purpose of enhancing health in dosages that exceed those that could be obtained from normal foods. These are sold in presentations similar to drugs: pills, extracts, and tablets.	[8]
	**5**. Numerous classes of compounds found in natural and processed foods that are claimed to have beneficial effects on human health and wellness, e.g., vitamins, carotenoids, flavonoids, curcuminoids, polyunsaturated fatty acids, proteins, peptides, dietary fibers, oligosaccharides, and minerals.	[9]
Functional food	**1**. A functional food is similar in appearance to, or may be, a conventional food, is consumed as part of a usual diet, and is demonstrated to have physiological benefits and/or reduce the risk of chronic disease beyond basic nutritional function	[6]
	**2**. The term “health/functional food” refers to food supplements containing nutrients or other substances (in a concentrated form) that have a nutritional or physiological effect whose purpose is to supplement the normal diet. The Korean Health/Functional Food Act from 2004 requires these products to be marketed in measured doses such as in pills, tablets, capsules, and liquids.	[10] (Korea perspective)
	**3**. A food that beneficially affects one or more target functions in the body beyond adequate nutritional effects in a way that is relevant to either an improved state of health and well-being and/or reduction of risk of disease: Not a pill, a capsule, or any form of dietary supplement Consumed as part of a normal food pattern	[11]
	**4**. Functional foods are the foods or dietary components consumption of which may have associated health benefits beyond the basic nutritional properties that the foods possess.	[8]

**Table 2:** European regulatory framework of food-related bioactive compounds.

Regulations			
Title	Year	Topic	Comments
Regulation (EC) No. 178/2002 of the European Parliament and of the Council of 28 January 2002 laying down the general principles and requirements of food law, establishing the European Food Safety Authority and laying down procedures in matters of food safety [13].	2002	General	Lays down the general framework of food law and safety; and establishes the European Food Safety Authority (EFSA).
Directive 2002/46/EC of the European Parliament and of the Council of 10 June 2002 on the approximation of the laws of the Member States relating to food supplements [15].	2002	Food Supplements	Focused on vitamins and minerals (*only vitamins and minerals listed in Annex I, in the forms listed in Annex II, may be used for the manufacture of food supplements*). Bioactive compound is marketed in a pre-packages form (pills, capsules, etc…). This regulation explicitly excludes any bioactive compound aimed to be used as a medicinal product.
Regulation (EC) No. 1925/2006 of the European Parliament and of the Council of 20 December 2006 on the addition of vitamins and minerals and of certain other substances to foods [16].	2006	Fortification of food	Includes vitamins, minerals and other substances added to food in order to enrich the food (*Annexes I and II are positive lists of vitamins and mineral, and their sources, while Annex III is a list of substances other than vitamins and minerals that are prohibited, restricted or under scrutiny*). This regulation also applies to food supplements (except for the provisions regarding vitamins and minerals).
Regulation (EU) No. 609/2013 of the European Parliament and of the Council of 12 June 2013 on food intended for infants and young children, food for special medical purposes, and total diet replacement for weight control and repealing Council Directive 92/52/EEC, Commission Directives 96/8/EC, 1999/21/EC, 2006/125/EC and 2006/141/EC, Directive 2009/39/EC of the European Parliament and of the Council and Commission Regulations (EC) No. 41/2009 and (EC) No 953/2009 [17].	2013	Foods for specific groups	Minerals, vitamins, amino acids, carnitine, taurine, nucleotides, choline and inositol that may be added to one or more of the categories of food for specific groups (*Annex is a single list of all substances that may be added to one or more of the categories of food for specific groups*).
Regulation (EU) 2015/2283 of the European Parliament and the council of 25 November 2015 on novel foods, amending Regulation (EU) No. 1169/2011 of the European Parliament and of the Council and repealing Regulation (EC) No 258/97 of the European Parliament and of the Council and Commission Regulation (EC) No. 1852/2001 [18].	2015	Novel foods	Food not used for human consumption to a significant degree at EU level before 15 May 1997. This regulation does not affect food enzymes, additives and flavorings, GMO, intended to be used in the production of foodstuffs or food ingredients.
Commission Implementing Regulation (EU) 2017/2470 of 20 December 2017 establishing the Union list of novel foods in accordance with Regulation (EU) 2015/2283 of the European Parliament and of the Council on novel foods [19].	2017	Novel foods	Novel foods authorized to be placed on the market within the Union as referred in Regulation (EU) 2015/2283 [18]. Contains Table 1*, with the conditions of use (including maximum levels), labeling and other requirements and* Table 2 *containing the specifications of the authorized novel foods*.

Note to the reader: All the regulations cited have several amendments and corrigenda that are not detailed for clarity reasons in the present review, however, the consolidated versions of the regulations have been consulted. Text in italics used for content of specific annexes/tables in the regulations.

**Table 3:** Tier approach suggested by the European Food Safety Authority (EFSA) for the safety evaluation of food additives (EFSA, 2012).

	Tier 1	Tier 2	Tier 3
Toxicokinetics (ADME)	Absorption studies and in vitro gastrointestinal metabolism	Studies to define distribution, metabolism and excretion and other basic toxicokinetic parameters following a single dose (OECD TG 417, [38])	Studies to define toxicokinetic parameters following repeated administration
Genotoxicity (based on [39])	- Bacterial reverse mutation assay (OECD TG 471, [40]) **and** - In vitro mammalian cell micronucleus test (OECD TG 487, [41]).	Follow-up of a positive results in Tier 1: - In vivo micronucleus test (OECD TG 474, [42]) **or** - In vivo Comet assay (OECD TG 489, [43]) **or** - transgenic rodent assay (OECD TG 488, [44]).	
Subchronic and chronic toxicity and carcinogenicity	Subchronic toxicity (at least 90 days) (OECD TG 408 [45]).	- Chronic toxicity (normally 12 months period) (OECD TG 452, [46]) **and** - Carcinogenicity (18–24 months period) (OECD TG 451, [47]), **or** - Combination of both studies (OECD TG 453, [48]) (Rat only)	Short-term tests with transgenic mouse models (p53+/−, rasH2, Tg.AC, Xpa−/− and Xpa−/−p53+/−) **and/or** Neurotoxicity, immunotoxicity or endocrine-mediated studies
Reproductive and developmental toxicity		*Effects on reproductive organs (Tier 1, subchronic toxicity):* - prenatal developmental toxicity study in rabbit (OECD TG 414, [49]) **and** - Extended One-Generation Reproduction Toxicity Study (EOGRTS) (OECD TG 443, [50])	Additional studies for e.g., endocrine, developmental neurotoxicity (OECD TG 426, [51]), and mode of action studies
	**To consider applying Tier 2:** - Systemic availability - Subchronic toxicity - Genotoxicity in vitro	**To consider applying Tier 3:** - Bioaccumulation - Genotoxicity in vivo - Chronic toxicity/carcinogenicity - Reproductive and developmental toxicity	
